# Highly Interactive Brain–Computer Interface Based on Flicker-Free Steady-State Motion Visual Evoked Potential

**DOI:** 10.1038/s41598-018-24008-8

**Published:** 2018-04-11

**Authors:** Chengcheng Han, Guanghua Xu, Jun Xie, Chaoyang Chen, Sicong Zhang

**Affiliations:** 1 School of Mechanical Engineering, Xi’an Jiaotong University, Xi’an, China; 2 State Key Laboratory for Manufacturing Systems Engineering, Xi’an Jiaotong University, Xi’an, China; 3 Department of Biomedical Engineering, Wayne State University, Detroit, Michigan USA

## Abstract

Visual evoked potential-based brain–computer interfaces (BCIs) have been widely investigated because of their easy system configuration and high information transfer rate (ITR). However, the uncomfortable flicker or brightness modulation of existing methods restricts the practical interactivity of BCI applications. In our study, a flicker-free steady-state motion visual evoked potential (FF-SSMVEP)-based BCI was proposed. Ring-shaped motion checkerboard patterns with oscillating expansion and contraction motions were presented by a high-refresh-rate display for visual stimuli, and the brightness of the stimuli was kept constant. Compared with SSVEPs, few harmonic responses were elicited by FF-SSMVEPs, and the frequency energy of SSMVEPs was concentrative. These FF-SSMVEPs evoked “single fundamental peak” responses after signal processing without harmonic and subharmonic peaks. More stimulation frequencies could thus be selected to elicit more responding fundamental peaks without overlap with harmonic peaks. A 40-target online SSMVEP-based BCI system was achieved that provided an ITR up to 1.52 bits per second (91.2 bits/min), and user training was not required to use this system. This study also demonstrated that the FF-SSMVEP-based BCI system has low contrast and low visual fatigue, offering a better alternative to conventional SSVEP-based BCIs.

## Introduction

Brain-computer interface (BCI) is a technology that bypasses the human’s normal peripheral-nerve pathways to intuitively control an external device using brain signals^1,2^. Using BCI technology, brain signals are acquired, processed, and encoded into commands to control external devices^3,4^. Electroencephalogram (EEG)-based BCI technology has been widely studied because it is non-invasive, simple to operate, and low cost^5,6^. Its use has been attempted for patient assistance, rehabilitation and many other fields. However, the majority of EEG-based BCI technologies are still in the stage of laboratory research, which has focused on the information transfer rate (ITR) and accuracy of interactive performance, while practical matters, such as user adaptation, comfortableness, and reliability, are less often considered.

The EEG-based steady-state visually evoked potential (SSVEP) BCI technology has been an interesting research target because of its excellent interactive potential^7^, such as high tolerance to artifacts and robust performance across users. Particularly, the SSVEP paradigm can achieve a very high ITR. In 2015, Gao Rong and others from China’s Tsinghua University designed an SSVEP-based spelling paradigm with a 5 × 8 stimulation matrix, which achieved an average ITR of 4.45 bps, demonstrating the potential for high interactive speed^8^. SSVEP BCI technology usually uses light-flashing methods or pattern reversal methods, wherein the changes in luminance contrast generate higher harmonic peaks in the processed EEG response^9–11^, which could improve accuracy after individual calibration. However, continuous high-intensity contrast changes are very uncomfortable and unfriendly to users and will cause visual fatigue easily^12,13^, or even induce migraines and seizures. In addition, in high-ITR BCI systems, harmonic peaks may not be favourable because more stimulation targets need to be assigned to more different frequencies, but the sensitive stimulation frequency range is narrow, and the overlaps of harmonics further restrict the stimulation frequency options. Conventional SSVEP stimulation methods have other interaction defects. For example, the performance of SSVEP systems is influenced by ambient light interference^14^, and stimulation targets interfere with each other. All of these factors restrict the application of SSVEP BCI.

Much research has been devoted to solving these problems. Polychromatic SSVEP^15^ or high-frequency SSVEP^16^ has been proposed to enhance comfort. However, all of these SSVEP methods are based on the perception of property changes in color space, flicker always exists, and the application scope is narrow. Similar to the perception of luminance contrast, motion perception is another basic function of the visual system^17^. The brain response can be elicited by the visual motion-onset paradigm, called the motion-onset visual evoked potential (mVEP), which typically contains P1, N2 and P2 components. Motion-onset visual evoked potential can provide a softer stimulus with reduced fatigue, are more stable and have strong components, especially for situations with smaller numbers of targets^18^. In 2008, Gao Rong first utilized translation-motion-elicited transient mVEPs to construct a BCI paradigm, with promising results^19^. In recent years, the use of mVEP paradigms in a variety of BCI systems has been attempted^20,21^. However, the features of mVEPs are mainly manifested in the time domain, the waveform is seriously masked in the strong-background EEG activities, complex recognition algorithms are required, and the contradiction between the number of targets and stimulus duration limit the ITR of mVEP paradigms.

In 2012, Xie used a method of oscillating expansion and contraction motions of Newton’s rings to elicit steady-state motion visual evoked potentials (SSMVEPs)^22^ and for the first time assessed the applicability of implementing SSMVEPs for BCI applications. The steady-state motion reversals were mildly stimulating, and the typical features of SSMVEPs were in the frequency domain, taking advantage of both mVEPs and SSVEP. Then, they further studied the effects of mental load and fatigue of the flickering stimulation and the Newton’s rings motion stimulation^23^. Under motion stimulation, the *α* power and ratio of *α* power to *θ* power were lower than the indicators of flash stimulation. In addition, after a long period of stimulation, the reduction in the response amplitude of motion stimulation was significantly less than the reduction for flashing stimulation. These results suggest that the SSMVEP paradigm has outstanding characteristics of low mental load and low visual discomfort for BCI applications. However, the Newton’s rings paradigm did not consider the local illumination variations of stimulation patterns, in which light twinkling was still coupled with motion stimulation, the light twinkling at the centre of targets reduced accurate perception, and the low screen refresh rate further increased the motion blur. The blurry motion paradigm could not reflect the inherent characteristics of SSMVEPs, which weakens the special advantages of SSMVEP BCIs.

The goal of this study is to implement a highly interactive BCI paradigm using flicker-free SSMVEP technology to offer a better alternative to conventional flicker stimulation methods. Our hypothesis is that the motion checkerboard stimulation method would keep uniform brightness at all local areas that delivered pure motion stimuli and that motion blur would be further reduced with a high-refresh-rate display to elicit SSMVEPs with a single frequency. The paradigm developed here had properties of low sensitivity to the change of contrast, no need for training, and less visual fatigue. The specific aims of this study were to develop a new ring-shaped motion checkerboard stimulation method and to test its efficiency for highly interactive performance as a paradigm of BCIs.

## Results

### FF-SSMVEP Stimulation

**Figure 1:**
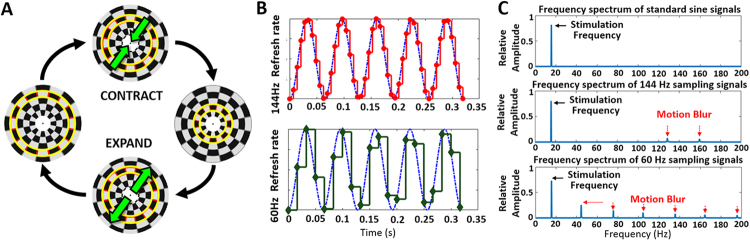
The design of the ring-shaped motion checkerboard paradigm. (**A**) The ring-shaped checkerboard stimulation pattern and the stimulation process of the paradigm. The expansion-contraction process of the motion checkerboard paradigm in one stimulus period is shown. The bright-yellow boundary line indicates the shape shifting of the same zone within different processes. The green arrows show the motion direction of the pattern texture. (**B**) Examples of stimulation signals under different refresh rates. The blue dotted line indicates the temporal waveform of theoretical stimulation signals. The frequency was set as 15.6 Hz. The red line indicates the stimulation signals that were sampled by the 144 Hz refresh rate. The green line indicates the stimulation signals that were sampled by the 60 Hz refresh rate. The fitting degree of the 144 Hz sampled signals was much higher than that of the 60 Hz sampled signals. (**C**) Examples of the frequency spectra of different sampling signals. The black arrows indicate the stimulation frequency. The red dotted arrows indicate the motion blur parts. The blur of the low refresh rate was more obvious as the stimulation frequency increased. At the same stimulation frequency, a high refresh rate provided highly accurate motion stimulation, closer to analytical values than the low refresh rate.

Our BCI design utilized a ring-shaped motion checkerboard pattern to realize FF-SSMVEP stimulation. Computer algorithms yielded a constant checkerboard size and a uniform luminance without light flashes being perceived by subjects. As shown in Fig. 1A, the static image of the stimulation pattern was composed of many concentric rings, and each ring was made up of white and black checks. The areas of white regions and black regions were set to be equal. Therefore, the overall brightness and local brightness of the pattern remained uniform, and the texture of the pattern was clear (see details in Materials and Methods). The checkerboard texture of the pattern motion was seen when the SSMVEP visual stimulation began. Figure 1A also shows the detailed expansion-contraction motion process, showing the change in the checkerboard pattern in one motion period. The bright yellow boundary line in Fig. 1A indicates the shape shifting of the same zone. The upper part of the figure indicates that all concentric rings continued to contract. The lower part of the figure indicates that all concentric rings continued to expand. When the outermost ring moved beyond the borders of the pattern, it disappeared, and a new ring appeared to supplement it; thus, the total size of the pattern remained unchanged. The motion process was modulated by a sinusoid function. The motion phase gradually shifted from 0 to *π*, so a contraction motion was presented; then, the phase gradually shifted from *π* back to 0, so an expansion motion was presented. Therefore, the motion direction changed twice in one cycle. Similar to SSVEPs, SSMVEPs are formed by a series of mVEPs over a steady-state stack. Since mVEPs are sensitive to the start of the motion, the SSMVEPs also mainly relied on the visual perception of motions oscillating in two opposite directions^24^. The peak energy of the SSMVEP response mainly focused on the motion direction change rate. For the presentation of such oscillating motion, each of the two motion directions would be presented half the cycle time and then be replaced by the other direction of motion, comprising one stimulus period. The direction change rate, which was specifically called the motion reversal frequency, served as the fundamental frequency of the SSMVEPs. The first subharmonic frequency equalled the motion cycle (sinusoid) frequency. A demo video of the stimulation has been uploaded in the supplementary material, it would be helpful to intuitively present motion stimulation.

The refresh rate of the display was another important factor to reduce the flickering feeling when presented with a motion process. Actually, all the motions presented on the display were discontinuous. Checkerboard motion stimulation was divided into discrete frames based on the monitor’s refresh interval. Due to the mechanism of persistence of vision, the human eye has the illusion of continuous motion. However, if the motion frequency increases, the number of frames used to present the motion decreases, motion blur increases, and the perceived light flashes increase accordingly. Motion blur occurs when retinal images of objects move relative to the retina^25,26^; if the motion is not continuous, a flashing feeling results. Particularly, under the low refresh rate of the monitor screen, the high-frequency motion stimulation could not be presented accurately. To solve the problem of a flicker feeling caused by confusion in motion rendering, a high-refresh-rate display is highly recommended for presenting motion stimulation. As shown in Fig. 1B and C, the sinusoidal stimulation signals presented on a high-refresh-rate (144 Hz) display and a low-refresh-rate (60 Hz) display were used to for comparison. The traditional display with a 60 Hz refresh rate did not produce clear waveforms in response to the stimulation at 15.6 Hz without distortion. Using the 144-Hz-refresh-rate display, the stimulation signal was similar to the theoretical value in both the time domain and the frequency spectrum (between 0 and 100 Hz), and the motion of ring-checkerboard was presented smoothly and stably without distortion.

### EEG data analysis outcomes

**Figure 2:**
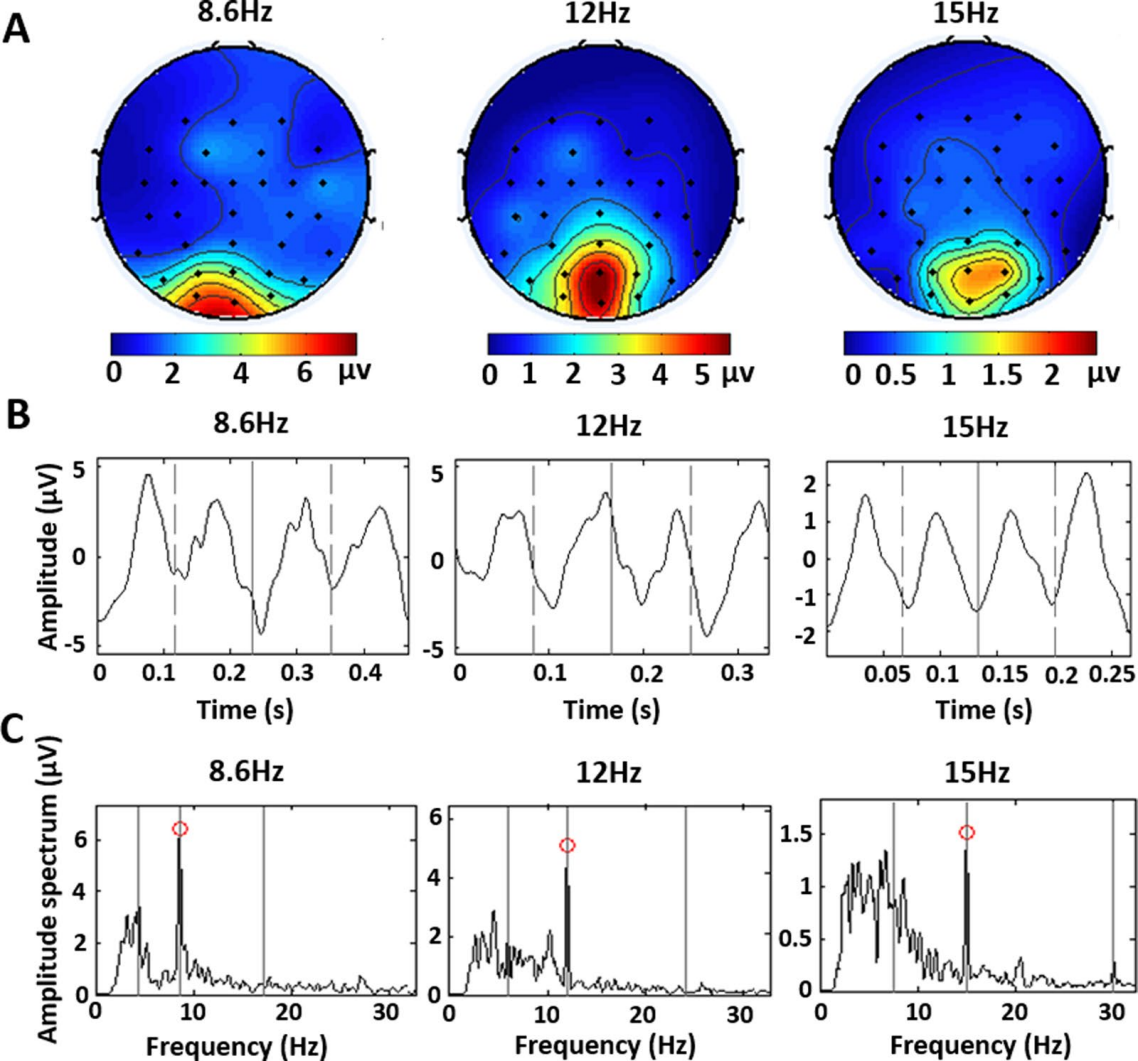
Distribution and time-frequency characteristics of SSMVEPs. (**A**) Figures on the top row show the brain electrical activity mapping with motion checkerboard stimulation acquired from 32 channels. The color indicates the amplitude of SSMVEPs with corresponding stimulation frequencies. The dots indicate EEG electrodes according to the 10–20 system. (**B**) Figures in the middle row show the time-domain signals of SSMVEPs that were acquired from the Oz channel. The vertical solid lines define a complete checkerboard motion, including both contracting and expanding periods. The vertical dotted line divides one complete checkerboard motion into two reverse motions corresponding to checkerboard contraction and expansion. (**C**) Figures on the bottom row show the amplitude spectrum of SSMVEPs. The red circles indicate the spectrum peaks of fundamental SSMVEP signals. The three vertical solid lines indicate the motion modulation frequency, motion reversal frequency, and second harmonic frequency of the stimulus.

As shown in the brain electrical activity mapping (Fig. 2A), SSMVEPs were mainly distributed symmetrically on the left and right sides of the occipital lobe and diffused into the parietal and temporal lobes. And the SSMVEP responses from the centre of occipital lobe were relatively strong. This indicated that double-direction alternate motion stimulation can cause symmetrical diffuse functional connections in the occipital lobe, activating a wide range of brain regions. In general, the signal of Oz (located in the centre of the primary visual cortex) had a high amplitude of visual evoked potentials, and the signal-to-noise ratio (SNR) of steady-state visual evoked response from Oz was relatively high^27^. A similar phenomenon of SSMVEP stimulation was presented on brain electrical activity mapping. Therefore, the average time-domain waveforms of SSMVEPs were obtained from Oz after digital signal processing. The results demonstrated that the time-domain waveform of SSMVEPs fit into a sine curve. The main signal period of SSMVEPs corresponded to the motion reversal period of the stimuli (Fig. 2B).

The highest spectrum peaks of SSMVEPs were accurately elicited in response to the motion reversal frequency of stimuli with a high SNR (Fig. 2C) (the second vertical line with red circles). At the point of the motion modulation frequency, the spectrum peaks could not be discriminated from the low-frequency background noise. At the point of the second harmonic frequency, only some less obvious spectrum peaks appeared. These results demonstrate that the frequency of SSMVEPs corresponded to the motion reversal frequency of the stimulus; only one peak was prominent in response to the motion reversal frequency. This SSMVEP signal had the properties of high time-frequency resolution and high energy concentration.

### Canonical correlation analysis (CCA)

**Figure 3:**
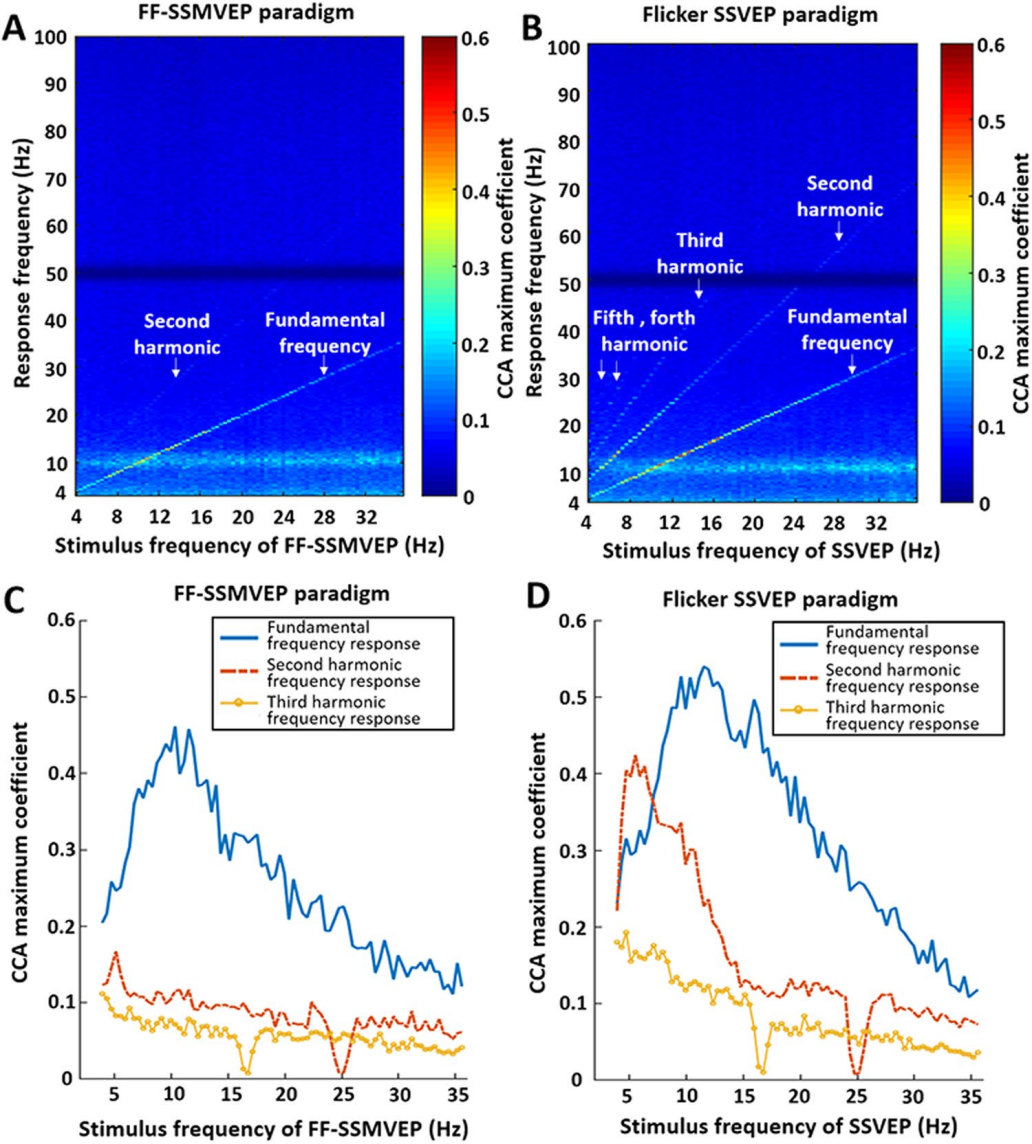
CCA coefficient spectrum and frequency response characteristics of the FF-SSMVEP and SSVEP stimulus paradigms. (**A**) CCA coefficient spectrum of the FF-SSMVEP paradigm. In the CCA coefficient spectrum, the vertical axis indicates the response frequency, the horizontal axis indicates the stimulation frequency, and the color indicates the value of the CCA coefficient. Under the stimulus of the FF-SSMVEP paradigm, the fundamental frequency response line was the only significant response. The second harmonic frequency response line was barely visible, and the higher harmonic frequency responses were almost invisible. (**B**) CCA coefficient spectrum of the SSVEP paradigm of flicker-evoked SSVEPs. Under the stimulus of the SSVEP paradigm, the fundamental frequency response line and higher harmonic frequency responses were all significant. (**C**) Fundamental, second harmonic and third harmonic frequency responses of the FF-SSMVEP paradigm. The horizontal axis indicates the stimulation frequency, and the vertical axis indicates the CCA coefficient under the corresponding stimulation frequency. Blue solid lines depict the fundamental frequency response. The red dotted line depicts the second harmonic frequency response. The yellow dotted line with circles depicts the third harmonic frequency response. (**D**) Fundamental, second harmonic and third harmonic frequency responses of the flicker SSVEP paradigm. The ratio of the CCA coefficient between the fundamental frequency response and second harmonic frequency response in the FF-SSMVEP paradigm was significantly less than the ratio in the flicker SSVEP paradigm.

Canonical correlation analysis can demonstrate the correlation intensity between EEG responses and SSMVEP stimulation. It is an important method of classification in visual BCIs. The CCA coefficient spectrum reflected the response characteristics under different stimulation frequencies. Fig. 3A and B, respectively, show the CCA coefficient spectrum under the SSMVEP paradigm and the SSVEP stimulus paradigm. Under FF-SSMVEP stimulation, only the fundamental frequency was visible in the coefficient spectrum, the second harmonic was barely visible, and the higher-order harmonics were completely invisible. However, the fundamental frequency and the higher harmonic frequencies were also visible in the coefficient spectrum under flicker SSVEP stimulation, and even the fifth harmonics response could be identified by a subtle line.

When stimulated by the motion checkerboard pattern (SSMVEP paradigm), the average CCA coefficients (4.0 Hz to 35.6 Hz) of fundamental, second harmonic and third harmonic frequency responses were 0.26, 0.08 and 0.06, respectively (Fig. 3C). The average CCA coefficients of the flicker SSVEP paradigm were 0.32, 0.16 and 0.08, respectively (Fig. 3D). The changing trend of the fundamental frequency response of the FF-SSMVEP paradigm was similar to that of the flicker SSVEP paradigm. The response amplitude reached the highest when the stimulation frequency was 7 Hz to 13 Hz and then dropped as the stimulation frequency increased or decreased. However, the second harmonic and third harmonic frequency responses elicited by the motion checkerboard pattern were significantly lower than those elicited by the flicker pattern or the Newton’s rings motion pattern.

In addition, a CCA comparison of the ratio of the fundamental frequency response to the multiple harmonic frequency response, which was elicited by the motion checkerboard FF-SSMVEP paradigm and the flicker SSVEP paradigm, was calculated through the T test. The normality and homogeneity of variance assumptions were satisfied, and the CCA coefficient between 48 Hz to 52 Hz was not involved in the calculations because of the effect caused by the notch filter. The ratio of the CCA coefficients between the fundamental frequency response and second harmonic frequency response in the motion checkerboard SSMVEP paradigm was significantly less than the ratio in the flicker SSVEP paradigm (T test, P = 0.0047 < 0.01). In addition, the ratio between the fundamental frequency response and the third harmonic frequency response in the motion checkerboard SSMVEP paradigm (with stimulation frequency less than 15 Hz) was also significantly less than the ratio in the flicker SSVEP paradigm (P = 0.0071 < 0.01).

### ITR and Accuracy

The BCI performance of the motion checkerboard pattern was verified by data from the 40-target BCI speller online experiments (see details in Materials and Methods). The CCA coefficient of the corresponding stimulus inversion frequency was significantly above the average, and the CCA coefficient of the corresponding fractional or multiple harmonic frequency was not significantly high. The SNR of the FF-SSMVEP response was high in the entire stimulus band. A direct CCA algorithm without a training process was applied to calculate the accuracy. The accuracy and ITR of all of the subjects in the 40-target online speller experiment are shown in Table 1. Each trial lasted 2 to 3.5 seconds, depending on the performance of the offline experiment. The average accuracy of all eighteen subjects was 94.00 ± 2.44%, the average ITR reached 1.52 ± 0.46 bps (91.2 ± 27.6 bits/min), and the highest ITR was 2.6 bps (156 bits/min). The highest ITR SSVEP paradigm was a 40-target speller of Gao Rong from China’s Tsinghua University.In recent years, The average ITR reached 4.45 bps. And the mean ITRs of the mVEP and SSVEP systems were 0.5 and 1.44 bps, respectively. Our experimental results demonstrate that the ITR of the FF-SSMVEP paradigm was above the average in the steady-state visually evoked potential-based BCI paradigms, achieved a relatively high speed.

**Table 1:** BCI performance of all subjects in the online experiment. Each trial lasted 2–3.5 s, including 0.5 s for gaze shifting. The testing data consisted of 6 blocks (40 trials).

Subject	Trial length, s	Accuracy, %	ITR, bps
S01	2.0	97.08	1.99
S02	2.5	95.83	1.62
S03	3.5	95.42	1.20
S04	3.5	90.00	1.08
S05	3.5	92.08	1.13
S06	3.0	92.92	1.31
S07	3.0	93.75	1.33
S08	2.0	98.33	2.04
S09	2.5	94.17	1.56
S10	2.5	92.08	1.50
S11	3.5	90.42	1.09
S12	3.0	93.75	1.33
S13	3.5	92.92	1.14
S14	2.0	99.17	2.60
S15	2.0	93.33	1.85
S16	3.5	94.58	1.18
S17	3.5	92.92	1.14
S18	2.0	93.33	2.31
Mean ± SD	2.83 ± 0.64	94.00 ± 2.44	1.52 ± 0.46

### Performance without training and antifatigue

**Figure 4:**
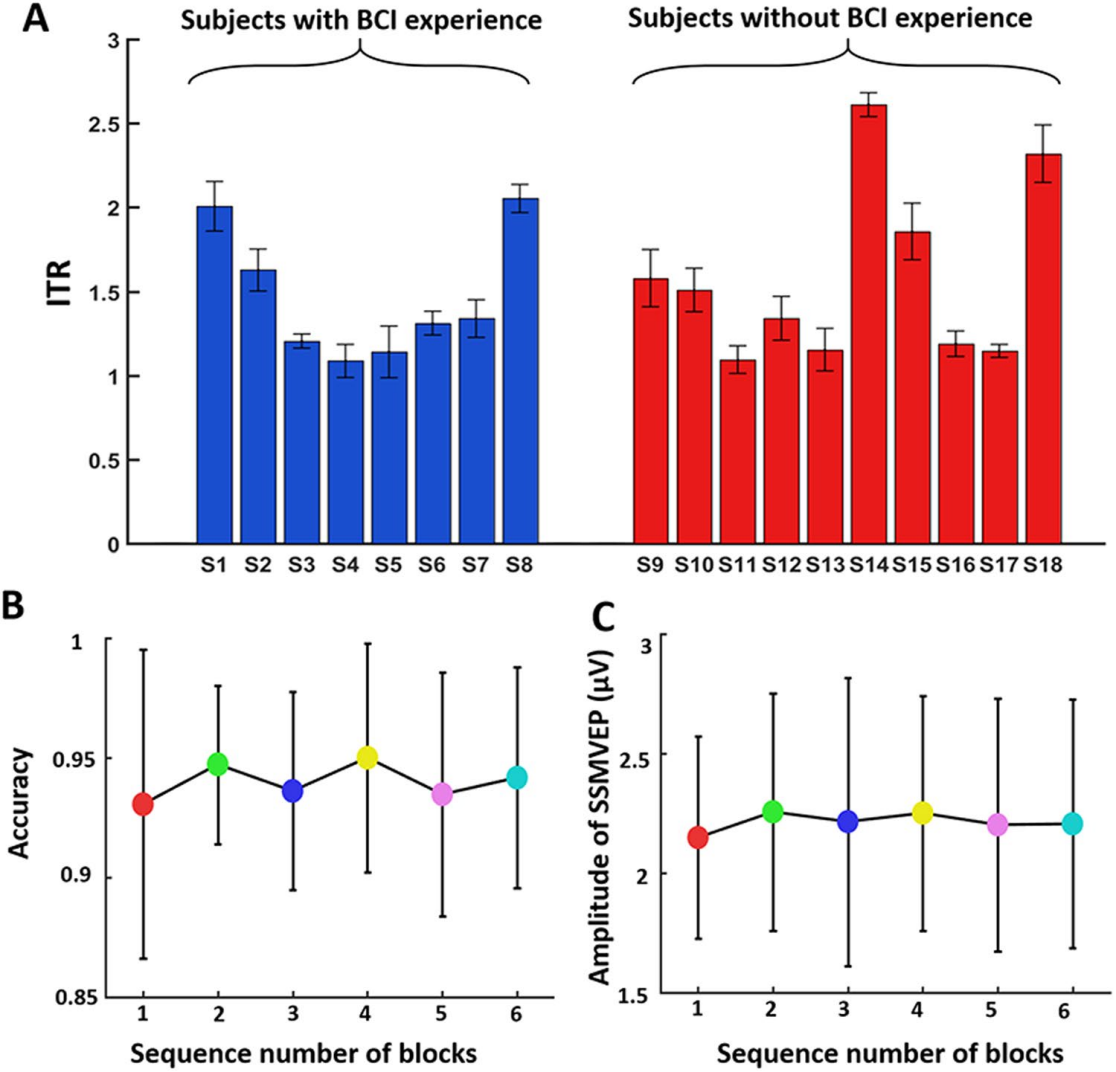
Non-training and antifatigue performance. (**A**) ITR of the groups with and without BCI experience. The difference between the two groups was not statistically significant. (**B**) The accuracy in each experimental block. (**C**) The change of amplitude in each experimental block. Six blocks of experiments were conducted continuously with a 1-min break, with each section lasting approximately 140 seconds, for a total time of approximately 20 minutes. The differences in accuracy and amplitude in all six blocks between groups were not statistically significant.

The subjects that participated in the online experiment were divided into the experienced group and the inexperienced group. The experienced group included eight subjects (S1 to S8) and the inexperienced group ten subjects (S9 to S18). The BCI performances of the two groups were analysed, and the ITRs of the two groups are presented in Fig. 4A. The ITR of the experienced group was 1.46 bps, and the ITR of the inexperienced group was 1.57 bps (P = 0.29).

In the online experiment, six sections of experiments were conducted continuously with a 1-min break. Each section lasted approximately 140 seconds, for a total time of approximately 20 minutes. The amplitude of SSMVEPs and the accuracy in all six sections were averaged because each frequency was not repeatedly represented. As shown in Fig. 4B, the amplitude of SSMVEPs and the accuracy were applied to reflect the level of fatigue. One-way analysis of variance (ANOVA) was used to test significance level. The results showed that the differences between the accuracies (P = 0.81) and amplitudes (P = 0.99) of the six blocks were not statistically significant, demonstrating that the FF-SSMVEP BCI performance did not decrease over time, thus indicating that the motion checkerboard stimulus caused little visual fatigue. Additionally, all of the subjects reported that the stimulation of FF-SSMVEPs was much more comfortable than the stimulation of SSVEPs, mainly because they did not feel any eye irritation.

### Performance of anti-interference under contrast change

The luminance contrast or “modulation depth” is an important property of visual stimulation. It is defined as the ratio between the sum of the brightness and the difference in brightness between light and dark areas in a pattern. When the luminance of ambient lighting increases, the contrast between the brightness of a stimulus and the background decreases. In offline experiments, motion checkerboard pattern with five levels of contrast were used to simulate different levels of interference from ambient light (see details in Materials and Methods).

**Figure 5:**
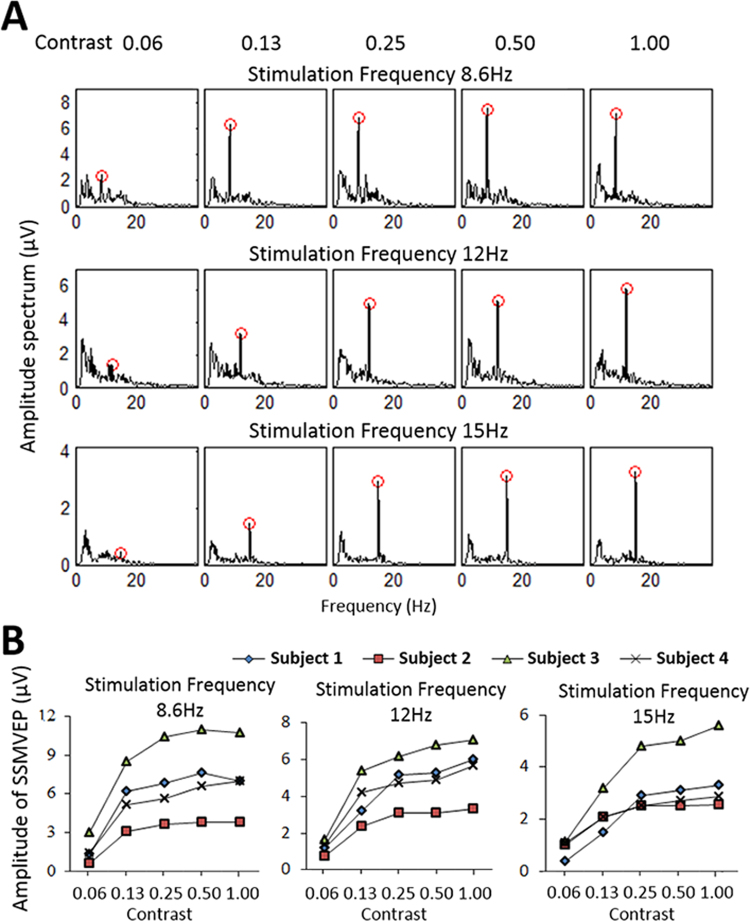
Performance variation of FF-SSMVEPs with stimulus contrast. (**A**) Average amplitude of SSMVEPs under different contrast stimuli and different frequencies (8.6 Hz, 12 Hz, and 15 Hz). When the contrast was greater than 0.25, the characteristics of the spectra from different frequencies of stimulation were not significantly different. The spectrum of the inversion stimulation frequency was obvious and had a very high SNR. (**B**) Amplitudes of SSMVEPs over all subjects under different contrast stimuli and different frequencies. The SSMVEP amplitude of all subjects to stimulating frequencies increased with increasing contrast from 0.06 to 1.00. The increase of SSVEP amplitude tended to be saturated at a contrast of 0.25.

The EEG data under the five contrast levels were bandpass filtered from 2 Hz to 22 Hz with a Butterworth filter and were separately averaged and computed by the FFT algorithm. When the contrast changed from 0.06 to 1.00, the amplitude of fundamental SSMVEPs gradually increased. The amplitude of responding SSMVEPs to increased contrast became larger but appeared to be saturated over a contrast of 0.25. Overall, contrast greater than 0.25 produced a very high SNR of fundamental SSVEPs (Fig. 5A). When the contrast was less than 0.25, the amplitude of fundamental SSMVEPs decreased significantly, and the SNR of signals also decreased significantly. The fitting curve of the amplitude was an ascending logarithmic curve. When contrast was greater than 0.25, the amplitude of SSMVEPs became saturated, with slow growth in response to contrast increments. The effect of different inversion frequencies appeared to be small over all subjects (Fig. 5B).

Considering the analysis above, the amplitude of SSMVEPs influenced by the stimulus contrast had no correlation with stimulation frequency (Pearson test, P > 0.05). The trends of SSMVEP amplitude changes were similar between subjects. The performance of the motion checkerboard stimulation was optimal when the contrast of the stimulus was greater than 0.25, independent of various lighting conditions. This demonstrated that the FF-SSMVEP BCI using the motion checkerboard stimulation had high anti-interference performance, with elicited SSMVEPs remaining stable in various lighting conditions.

## Discussion

Flicker stimulation is the most common way to induce a visual response in BCI research. VEP paradigms, mVEP paradigms, SSVEP paradigms, and even a Newton’s rings SSMVEP paradigm can be coupled to brightness modulation. However, flicker stimulation is uncomfortable for users. How to maintain the technical advantages while eliminating the flickering feeling is an important research direction of visual BCI research. In this study, a FF-SSMVEP paradigm based on a ring-shaped motion checkerboard was proposed to elicit SSMVEPs. The ring-shaped checkerboard was composed of a series of concentric rings of equal width, each ring divided into black and white squares of the same size and number. This ensured that the brightness contrast of targets would always be constant when the texture expanded and contracted. The motion stimulation was presented in a high-refresh-rate display, further reducing the flicker. The FF-SSMVEP paradigm thus decoupled the influence of brightness changes from the stimulation, presenting pure and mild motion visual stimulation, giving it high potential for application.

The FF-SSMVEP paradigm elicited concise fundamental SSMVEPs with little harmonic response. Since SSMVEPs mainly rely on the visual perception of motions oscillating in two opposite directions, the powers of SSMVEPs mainly reflect motion inversion frequency (the motion direction change rate). Therefore, the motion inversion frequency elicited the exact fundamental frequency of SSMVEPs. In contrast, the SSVEPs usually have higher harmonics. Although higher harmonics can improve the classification accuracy of an SSVEP system that contains smaller targets^28^, disturbances occurred when the interval of stimulation frequencies decreased and the number of stimulation frequencies increased. Limited by the maximum sensitive stimulation frequency range, which is 5 Hz to 13 Hz^29^, the selectable frequencies have to be chosen carefully to ensure non-overlap of SSVEP responses in a multiple-target SSVEP BCI system. Our flicker-free SSMVEP paradigm could provide a new approach to minimize the problems mentioned above. Although the average amplitude of SSMVEPs was slightly less than that of SSVEPs, SSMVEPs had the advantages of energy concentration and concise frequency. The spectrum of SSMVEPs only had a single significant peak, corresponding to the motion inversion frequency, making the response feature stand out from the EEG background. Due to this property, potential harmonic overlaps of multi-frequency stimuli were avoided, and the stimulation frequency for selection from the narrow sensitive range increased. Comparing the CCA coefficients for SSVEP with SSMVEP, SSMVEP has lower values, which means the SSVEP has a slightly higher SNR. However, the strongest contrast (pure black and pure white) were used in SSVEP experiments, which means the intensity of stimulation can no longer increase. The SSMVEP paradigm can further improve the response intensity by optimizing the amplitude of motion stimulation, the application potential is stronger.

The ITR is an important criterion for BCI evaluation. In the FF-SSMVEP online experiment, a 40-target speller was designed and utilized to test the speed of the system. The results show that the average ITR of FF-SSMVEP was 1.52 ± 0.46 bps, the average accuracy was 94.00 ± 2.44%, and the mean time to issue a command was 2.83 ± 0.64 s. By comparison, the mean ITR of BCI systems in recent years is 0.94 bps. Specifically, the mean ITR for mVEP and SSVEP systems is 0.5 and 1.44 bps, respectively^30^. These data demonstrate that the FF-SSMVEP-BCI system had higher speed indicators than the average SSVEP-BCI system and greatly improved the ITR of the BCI system based on motion visually evoked potentials. The improvement in performance can be attributed to the concise frequency feature because many optimal stimulation frequencies can be arbitrarily assigned regardless of harmonic interference. The recognition algorithm was also simplified, providing the possibility of instant use.

The highest ITR SSVEP paradigm was a 40-target speller of Gao Rong, the average ITR reached 4.45 bps. The average ITR of the FF-SSMVEP paradigm we proposed reached 1.52 ± 0.46 bps (91.2 ± 27.6 bits/min), the speed indicator at a disadvantage. However, the SSVEP-based BCI paradigm of Gao Rong required complex machine learning classifier and synchronize hardware. A large area of flashing also was quite uncomfortable. When fatigue occurred, important parameters of response signal such as phase would change, reduce system robustness. Compared with the highest ITR SSVEP paradigm, the FF-SSMVEP-based BCI paradigm we proposed only required household high-refresh-rate monitor, mild stimulation, high robustness, the number of stimulation target could be further enhanced, had better potential applications in BCI rehabilitation and other fields.

The interactive performance of the flicker-free SSMVEP paradigm yielded satisfactory results outside of speed. The quality of the interactivity, such as user adaptation, comfort and reliability, has become increasingly important in the practical utilization of BCI technology^31^. In this study, a total of eighteen subjects participated in the online experiment. Among them, eight subjects had visual evoked potential experiences before, and ten subjects participated in a BCI experience for the first time. There was no significant difference in ITRs between the experienced group and inexperienced group. This indicated that a training factor was not important in the performance of the SSMVEP system. Inexperienced participants smoothly operated the online system with a training-free CCA algorithm. Thus, the FF-SSMVEP paradigm had satisfactory performance in user adaptation.

Compared with lightness contrast stimulation, the flicker-free motion stimulation is much more comfortable and causes less fatigue. A fatigue analysis was performed for quantitative evaluation. In the online experiment, the average time of each block was 130 seconds. Six blocks were executed sequentially, for an average time of the online experiment of approximately 20 minutes. The results indicated that the accuracy and ITR did not change with prolonged experiment time. All subjects reported that the motion checkerboard stimulation was more comfortable than the traditional flicker stimulation. However, some participants reported when they gradually perceived flicker when stimulation frequency higher than 25 Hz, and the motion perception become increasingly weaker. So, we assume the paradigm (presented in 144 Hz refresh-rate display) was flicker-free when the stimulation frequency lower than 25 Hz, and a higher refresh-rate display could alleviate this restriction. This demonstrates that the FF-SSMVEP paradigm was a mild visual stimulation that elicited SSMVEPs that were stable and caused less visual fatigue, in addition to good performance for a long period of time.

The performance of anti-interference under contrast change of FF-SSMVEPs was excellent. The visual perception of motion information was not vulnerable to the change of contrast. Due to this characteristic, the SSMVEP paradigm remained stable when the contrast of the stimulus changed. In contrast, SSVEPs can be significantly affected by ambient light^14^: the depth of contrast modulation of stimulation targets decreases when the luminance of ambient light increases. For this reason, the amplitude of visually evoked potentials becomes unstable, and the robustness of the BCI system decreases. Therefore, the modulation depth method of the SSVEP paradigm usually is set to bright white or bright red to contrast with black^32^, with the luminance contrast set at above 0.5 to ensure that the flicker is perceived steadily. However, our offline experiment demonstrates that the amplitude of SSMVEPs remained stable when the contrast of the stimulus decreased from 1.00 to 0.25, only presenting a mild descending tendency. The amplitude of SSMVEPs significantly decreased only when the contrast decreased below 0.13. The contrast threshold of the SSMVEP method was lower than that of the traditional SSVEP flicker method.

The FF-SSMVEP paradigm based on a ring-shaped motion checkerboard completely decoupled the changes of brightness and motion, enabling it to elicit remarkable fundamental frequency responses without multiple harmonic frequency responses. This feature increased the alternative stimulation frequency selection in the BCI system for visual stimuli, avoided interference, and increased the accuracy of performance. This FF-SSMVEP paradigm does not need user training or complex learning algorithms, is more comfortable, and causes less visual fatigue. In laboratory testing and practical testing, the FF-SSMVEP BCI demonstrated its advantages compared to traditional visually evoked potential methods, exhibiting a high performance in man-computer interaction. Meanwhile, the ring-shaped motion checkerboard accurately reflected the contracting and expanding motion process, which provides a new avenue for research on motion perception.

## Methods

### Stimulation Setup

Our BCI design utilized the motion checkerboard patterns to construct visual stimulus and displayed them on a Philips 242G5DJEB 27-inch liquid-crystal display screen (1,920 × 1,080 pixels resolution and 144 Hz refresh rate). The outer diameter of motion checkerboard pattern in our experiment was set at 100 pixels and the inner diameter was set at 20 pixels. The motion checkerboard pattern consisted of eight concentric rings, each ring was divided into 16 equal sectors of black and white squares. The amplitude of expansion-contraction motion process was set at 5 to 15 pixels. The width of one pixel was 0.31 mm, subjects sat about 80 cm away from the LCD monitor. The visual angle of motion checkerboard was 100 × 0.31 / 800 × (180 / 3.14) = 2.22°. The motion checkerboard pattern was developed using MATLAB and the Psychophysics Toolbox Version 3^33^.

### Offline experiment

**Figure 6:**
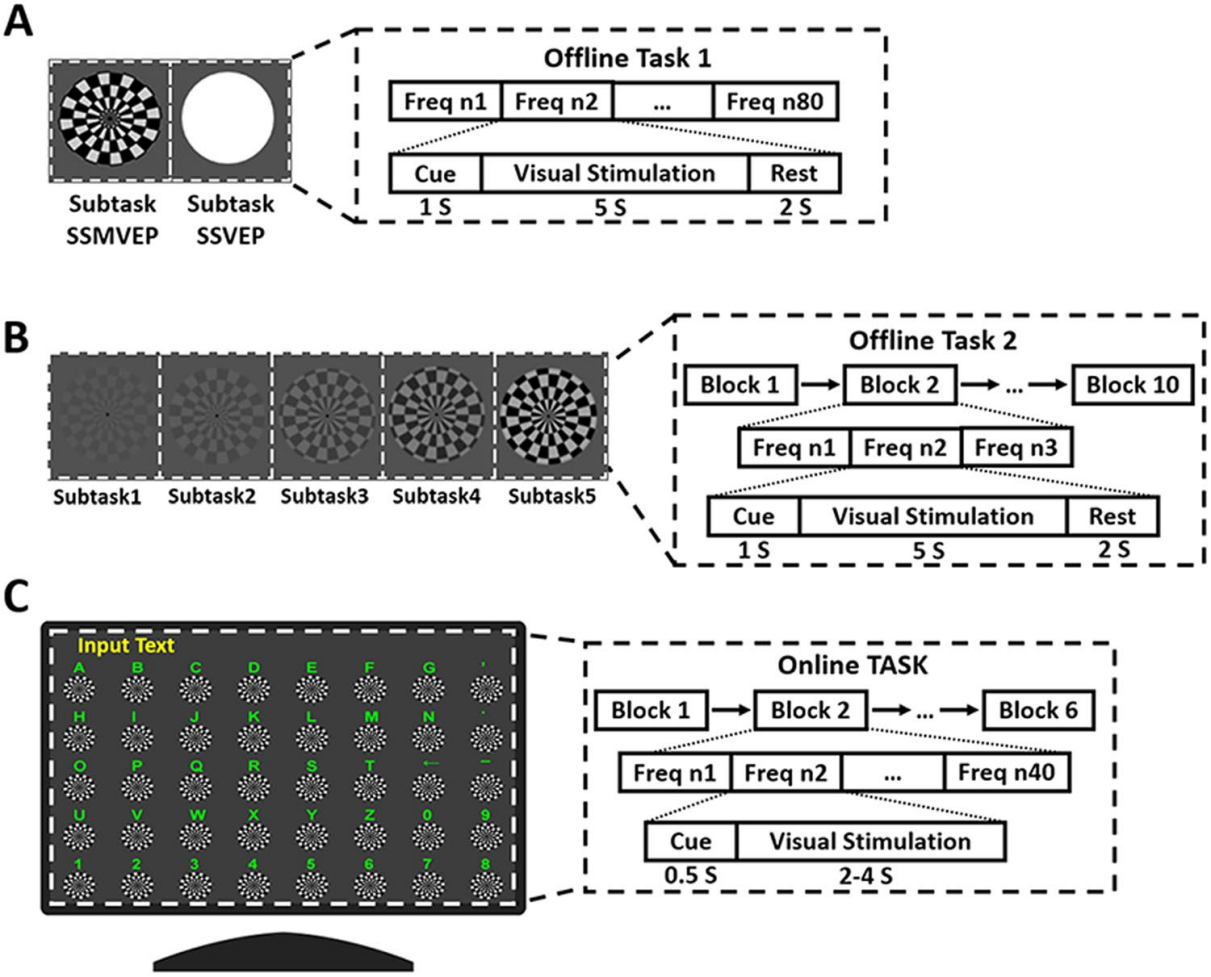
Sketch of offline experiment and online speller experiment. (**A**) In offline experiment task 1, subjects were asked to gaze at the motion checkerboard (subtask 1) or a flicker target (subtask 2). Each subtask contained 80 trials, with each trial lasting 8 s. Each trial began with a visual cue, which appeared for 1 s in the centre of the screen. Following the cue’s disappearance, one stimulus with random frequency began to produce motion or flicker on the screen, lasting 5 s. After the stimulus, the screen was blank for 2 s before the next trial began. Each subtask last for 640 s. There was a rest for several minutes between two subtasks to avoid visual fatigue. (**B**) The process of task 2 was similar to that of task 1. The subtask included six blocks (3 trials), with each trial lasting 8 s. Each trial began with a visual cue that appeared for 1 s. Then, one stimulus began to move and lasted 5 s, followed by 2 s of a blank screen for resting. Each subtask last for 240 s. There was a rest of several minutes to avoid visual fatigue. (**C**) The online BCI speller consisted of 40 visual stimuli, which were arranged in an 8 × 5 stimulation matrix resembling an alphanumeric keyboard. Each stimulus represented a unique alphanumeric character, punctuation character or manipulated character. Each stimulus was rendered within a 100-pixel-diameter ring. The vertical and horizontal distances between two neighbouring stimuli were 100 pixels. The online task included 6 blocks (40 trials corresponding to all 40 targets), with each trial lasting 2.5–4.5 s, including 2–4 s for visual stimulation and 0.5 s for gaze shifting. There was a 1-min break after each block. The environment of the online experiment was consistent with that of the offline experiment.

The offline experiment consisted of two tasks. Task 1 focused on the time-domain and frequency-domain characteristics of SSMVEPs. Task 2 focused on the influence of stimulation contrast. Experiments were performed in a quiet and ordinarily lighted room with no electromagnetic shielding. All subjects were requested to sit on a comfortable armchair, approximately 80 cm in front of the LCD monitor. They were asked to stare at the target stimulator and not to track the movement of the stimulator with their eyes. In task 1, subjects were asked to gaze at the motion checkerboard (subtask 1) or a flickering target (subtask 2). The motion inversion frequencies or flicker frequencies of stimuli were set from 4.0 Hz to 35.6 Hz with increments of 0.4 Hz. Each stimulus was applied within a 100-pixel-diameter ring. Eighty trials corresponding to 80 frequencies were performed. The minimum or maximum brightness of the flicker and the motion checkerboard were equal (see details in Fig. 6A). Task 2 consisted of 5 subtasks. In each subtask, one motion checkerboard was presented in the centre of the screen with a different contrast (0.13, 0.17, 0.22, 0.26 or 0.30 degrees). Contrast was calculated as follows: c = (lmax − lmin)/(lmax + lmin), where lmax is the highest brightness, lmin is the lowest brightness, and c is the contrast value^34^. Subjects were asked to gaze at a motion checkerboard. The motion inversion frequency was set at 8 Hz, 10 Hz or 12 Hz. Every block presented the motion checkerboard with the three frequencies in random order (see details in Fig. 6B).

### Online BCI speller

An online BCI speller was proposed to demonstrate the BCI performance of the motion checkerboard paradigm. In the online BCI speller experiment, the subjects were asked to perform a cued spelling task. The motion inversion frequency of stimuli was 7.0 Hz to 14.8 Hz with increments of 0.2 Hz. When the experiment began, a character was shown in boldface as a cue to indicate the target. Subjects were asked to shift their gaze to the motion checkerboard pattern below the character as soon as possible within the cue duration. Following the cue offset, all stimuli were presented on the screen and concurrently moved. When the stimulus motion stopped, the character result was typed on the top of the screen as visual feedback. At the same time, the next cue (no duplicate) was shown in boldface for the following trial (see details in Fig. 6C).

### Participants

Eighteen healthy subjects (5 females; aged 20–25 years, mean age 22 years), with normal or corrected-to-normal vision, participated in the experiments. All of eighteen subjects participated in the online experiment. Among them, eight subjects (S1–S8) had visual evoked potential experiences before. The other ten subjects participated in a BCI experience for the first time, they were entirely unfamiliar with the BCI system. The subjects in experienced group had participated in the SSMVEP online experiments several times. We chose the trial time based on their relevant training data before. And for subjects in inexperienced group, before the formal experiments, each of them was asked to perform online spelling task for three times to become familiar with experiment process. The trial time is gradually reduced from 3.5 seconds to 2 seconds, we selected the shortest possible trial time which could maintain the accuracy greater than 90

### EEG Data Recording

EEG signals were recorded with a g.USBamp (g.tec Inc., Austria) from 32 EEG electrodes (Fz, F3-4, FC1-2, FC5-6, Cz, C1-6, CPz, PC3-6, Pz, P3-4, POz, PO3-4, PO7-8, Oz, O1-2) according to international 10–20 electrode system. The reference channel was set at unilateral earlobe (A1), and the ground channel was set at frontal position (Fpz). The EEG signals sampled at 1200 Hz, band-pass filtered from 2 to 100 Hz and notch filtered between 48 to 52 Hz. All the electrode impedances were kept below 5 k Ω. In online experiments, only six EEG channel (Oz, O1, O2, PO1, POz, PO2) were used. This set-up was used throughout all the experiments. In the online experiment, EEG data were recorded and analyzed by the online data analysis program in real time. The online data analysis program was developed under MATLAB (MathWorks, Inc.).

### Data Preprocessing and Time-frequency Characteristics Analysis Method

In the offline and online experiments, data epochs were extracted according to event triggers generated by the program. Considering a latency delay in the visual system^35^, EEG data epochs were extracted with a 150-ms delay, which had the highest classification accuracy across all subjects. All epochs were bandpass filtered from 2 Hz to 100 Hz (Butterworth filter). Notch filtering between 48 and 52 Hz was implemented using the amplifier API in MATLAB. The recorded EEG data under the motion checkerboard stimulation were analysed offline to determine time-frequency characteristics. Since the Oz is located at the centre of the primary visual cortex and usually has the stronger evoked potential amplitude, recorded EEG data from the Oz were analysed. In time-domain analysis, the 5-s-duration offline EEG epochs were divided into non-overlapping data segments, with each segment containing two periods of the contraction-expansion stimulation process, and then after average superposition, the time-domain waveforms of SSMVEPs were obtained. In frequency-domain analysis, each EEG epoch was weighted by the Hamming window function. After FFT transform, the amplitude spectra of SSMVEPs were obtained.

### CCA-Based Target Identification Method

Canonical correlation analysis (CCA) has been successfully applied for frequency detection in multi-channel SSVEP-based and SSMVEP-Based BCIs^22,36^. It is a multivariable statistical method to calculate the underlying correlation between two sets of variables. A CCA recognition algorithm without machine learning was implemented to extract frequency components and detection targets. The online experiment target identification process included first calculating the CCA coefficients between the EEG signal and the template signal of all stimulation frequencies and then selecting the stimulation frequency that corresponded to the maximum coefficient as the final recognition result. Suppose that there were N stimulation inversion frequencies f1, …, fN in the system, for detecting the stimulation target, two sets of signals should be introduced into CCA. One set is the EEG signals X, from several different recording channels. The other set is stimulation signals Yi (i = 1, …, N), denotes the reference signals, is constructed as

1 $${Y}_{i}=(\begin{array}{c}sin\mathrm{(2}\pi {f}_{i}n)\\ cos\mathrm{(2}\pi {f}_{i}n)\end{array})\,\,n=\frac{1}{Fs},\frac{2}{Fs}\mathrm{,...,}\,\frac{K}{Fs}$$

where Fs is the sampling rate, and K is the number of sampling points. In our system, the data from six EEG channel which placed at occipital areas (Oz, O1, O2, PO1, POz, PO2) were used for CCA calculation. The response frequency of SSMVEP, which elicited by the motion checkerboard pattern, contained motion inversion frequency mainly, and few higher harmonics were contained, therefore, the reference signals Yi were only composed of sinusoid and cosinusoid pairs at the same frequency of the stimulus. CCA can find a pair of weight vectors Wx and Wyi to maximize the canonical correlation between linear transformations x = XT Wx and y = YiT Wyi by the following optimization problem:

2 $$\mathop{{\rm{\max }}}\limits_{{W}_{x},{W}_{yi}}\rho (x,{y}_{i})=\frac{E[{W}_{x}^{T}X{Y}_{i}^{T}{W}_{yi}]}{\sqrt{E[{W}_{x}^{T}X{X}^{T}{W}_{x}]E[{W}_{yi}^{T}Y{Y}_{i}^{T}{W}_{yi}]}}$$

The maximum of *ρ* corresponds to the maximum canonical correlation between X and Y _*i*_. When each canonical correlation of f _*i*_ (i = 1, …, N) was calculated separately, the target could be judged by the maximum *ρ* of N canonical correlations.

### Performance Evaluation

Information Transfer Rate (ITR) was used to evaluate the online performance of the BCI system. ITR is an important criterion to describe the detection accuracy and the time required of BCI system. In this study, the ITR in bits/min was adopted to evaluate the performance of the BCI system^37^. ITR were calculated as follows:

3 $$ITR=\frac{60}{T}[lo{g}_{2}N+plo{g}_{2}p+\mathrm{(1}-p)lo{g}_{2}(\frac{1-p}{N-1})]$$

where N is the number of targets (i.e., 40 in this study), p is the mean accuracy over all targets and T is the decision transfer interval (the sum of stimulation time and prompt interface presentation time).

### Data availability

The datasets generated and/or analyzed during the current study are available from the corresponding author on reasonable request.

## Electronic supplementary material

Supplementary Video
